# Effects of I125 seed stent implantation combined with arterial infusion chemoembolization on tumor markers, p53 expression, and prognosis in patients with cholangiocarcinoma

**DOI:** 10.1080/21655979.2021.2022263

**Published:** 2022-01-17

**Authors:** Zhe Xu, Minglin Xu, Meng Li, Zhe Cui, Haisheng Bai, Heqing Shang, Jianyu Zhao, Lei Wu, Fan Zhang

**Affiliations:** a Department of Interventional Therapy, Hulunbuir People’s Hospital, Inner Mongolia China; b Department of Reproductive Center, Hulunbuir People’s Hospital, Inner Mongolia China

**Keywords:** I125 particle stent, cholangiocarcinoma, arterial infusion chemoembolization

## Abstract

Cholangiocarcinoma is a common malignant tumor. Advanced treatment is difficult and the prognosis is poor. It is of great significance to find an effective method to treat cholangiocarcinoma and improve the prognosis of patients. Therefore, 78 patients with cholangiocarcinoma treated in our hospital were divided into group A and group B according to different treatment methods. The clinical effect, bilirubin, tumor size, bile duct patency time, tumor marker level To evaluate the therapeutic effect of I125 seed stent implantation combined with arterial infusion chemoembolization in patients with cholangiocarcinoma. The results showed that I125 seed stent implantation combined with TACE in the treatment of patients with cholangiocarcinoma can play an obvious clinical effect, effectively reduce the level of tumor markers and p53, reduce tumor lesions, improve the survival rate of patients, and play an important role in tumor treatment.

## Introduction

Cholangiocarcinoma is a common malignancy with an extremely low morbidity rate, so the patients tend to ignore the disease at the early stage and do not see a doctor. What’s worse, they miss the best cure time, because cholangiocarcinoma has reached the advanced stage over time [1]. Due to complex disease conditions, it is relatively difficult to radically treat or resect the advanced-stage cholangiocarcinoma, and the complications and spread of lesions will be triggered postoperatively. Therefore, special attention should be paid to whether various factors in surgical treatment for cholangiocarcinoma will affect the prognosis of patients, and then the treatment regimen is optimized based on the certainty factors, thereby evaluating the uncertainties in treatment more accurately and reasonably [2]. Remarkable achievements have been made in the molecular biology of cholangiocarcinoma in recent years, which greatly helps analyze and research the cure of this disease and can be directly applied to guide the clinical practices, but the research of tumor markers remains stagnant without novel prominent advances, which is also associated with the complexity of cholangiocarcinoma lesions. Currently, there have been a few drugs available for improving the prognosis of cholangiocarcinoma, bringing large difficulties to clinical treatment. Studies have found that after transcather arterial chemoembolization (TACE) treatment, tumor cells promote the production of tissue angiogenesis factors under hypoxia, which can stimulate tumor angiogenesis and tumor recurrence and metastasis [3]. TACE is greatly helpful for the clinical treatment of cholangiocarcinoma, and it is also one of the most efficacious measures to relieve biliary obstruction in treating cholangiocarcinoma [4]. Iodine-125 (I-125), a radioactive nuclear element, can emit radioactive rays to promote the apoptosis of cancer cells, and radioactive I-125 seed-based stent is widely applied to the treatment of tumors, since it is characterized by precise positioning and small trauma and can effectively resist the progression of lesions and even lessen them [5]. I-125 seed-based stent implantation combined with TACE can prevent the progression of tumors, thereby prolonging the survival of patients [6]. However, its related role in cholangiocarcinoma is still unclear. Therefore, we hypothesize that i125 seed stent implantation combined with TACE treatment of patients with cholangiocarcinoma can have a certain impact on the tumor markers, P53 expression, and prognosis of patients. Therefore, this article selects patients with cholangiocarcinoma undergoing treatment in our hospital and gives i125 seed stent implantation combined with TACE treatment. The aim is to explore its influence on the tumor markers, P53 expression, and prognosis of patients with cholangiocarcinoma, and to provide a relevant theoretical basis for the clinical treatment of cholangiocarcinoma.

## 1. Information and methods

### 1.1 Study subjects

The clinical data of 78 patients with cholangiocarcinoma admitted to our hospital from March 2016 to February 2018 were collected, and 78 patients were randomly divided into group A according to the different treatment methods of the patients (39 patients with biliary tract implanted with i125 radioactive seed stent), group B (39 patients with biliary tract implanted with i125 radioactive seed stent combined with TACE). The area of tumors and the obstruction position in the two groups of patients were first determined via imaging examinations, and then the radioactive I-125 seed-based stent was implanted according to the angiography through the external drainage tube. At 2 weeks postoperatively, TACE was performed in each cholangiocarcinoma patient for 3 times on average and 117 times in total when their liver function got restored well. The clinical information of patients in the two groups was collected, and there were no statistically significant differences in the sex, age, and tumor metastasis and staging between the two groups (*p* > 0.05) (Table 1). Prior to operation, the patients were subjected to percutaneous liver puncture biopsy for confirming the diagnosis, and they were evaluated and signed the consent to interventional therapy.

**Table 1.** Patient details

Basic information	Group A	Group B	*χ^2^*	*P*
Gender			0.217	0.641
male	25(64.10%)	23(58.97%)		
female	14(35.90%)	16(41.03%)		
age/year			1.902	0.386
<40	3(7.69%)	5(12.82%)		
40–70	28(71.79%)	30(76.92%)		
>70	8(20.51%)	4(10.26%)		
transfer			1.169	0.279
Intrahepatic	17(43.59%)	11(28.21%)		
Extrahepatic	8(20.51%)	10(25.64%)		
Cholangiocarcinoma			0.889	0.641
StageI	10(25.64%)	8(20.51%)		
StageII	22(56.41%)	26(66.67%)		
Stage III	7(17.95%)	5(12.82%)		

### 1.2 Diagnosis criteria

Inclusion criteria: patients definitely diagnosed with cholangiocarcinoma through AFP, computed tomography (CT), and magnetic resonance imaging (MRI) examinations and pathological puncture, those conforming to the criteria in the *Guidelines for Diagnosis and Treatment of Primary Liver Cancer* and *Regulations on the Standardization of Comprehensive Interventional Treatment of Liver Cancer*, and those with the ECOU score <2 points, and KPS score >70 points. Approved by the hospital ethics committee (ethics approval number: 2016-KY-044-01).

Exclusion criteria: patients complicated with other malignant tumors or major diseases, those who used other anti-tumor drugs, those with recurrent cholangiocarcinoma, those suffering from organ failure, or severe intrahepatic bile duct stenosis or extrahepatic bile duct obstruction, or others who were unable to receive the biliary stent implantation.

### 1.3 Treatment Methods

Collect 3 ml of venous blood from patients with cholangiocarcinoma before surgery and send it for examination in time. After centrifugation and separation of the blood sample, the blood sample was taken out and analyzed with a chemiluminescence analyzer [7] to analyze the tumor markers carbohydrate antigen 19–9 (Carbohydrate Antigen 19–9, CA19-9), carbohydrate antigen (Carbohydrate Antigen242, CA242) and the expression level of carcinoembryonic antigen (CEA), followed by puncture for sampling and biliary drainage. Under the monitoring by the DSA equipment, the puncture route was confirmed based on CT, MRI, and ultrasonography, and when the patients breathed quietly and the puncture successfully accessed to the biliary tract, the external drainage tubes were implanted into the left and right intrahepatic bile ducts to observe whether jaundice abated. When jaundice abated, the radioactive I-125 seed-based stent was implanted based on the classification of patients by the Bismuth-Corlette Anatomic Classification System, namely the stent was placed in the right intrahepatic bile duct of the type IIIA patients, with the external drainage tube in the left intrahepatic bile duct, while the procedures were the opposite in those with type IIIB cholangiocarcinoma. After implantation of the radioactive I-125 seed-based stent, angiography needed to be conducted for review monthly, which could be used to observe the stent and patency better. At 2 weeks after operation, the recovery of liver function of the patients in Group B was observed, and when the liver function was restored to grade A, TACE was conducted.

Western blot method [8] was used to detect the expression level of P53: add goat anti-human P53 monoclonal antibody, incubate with 37 antibody for 1 to 2 h, wash 3 times with PBST, 10 min each time, analyze the expression level of P53.

### 1.4 Efficacy evaluation and follow-up

The evaluation criteria for the treatment of tumors are as follows: whether the tumors completely disappear, and shrink by over 50% in area and the expression levels of tumor markers and P53 are lowered. At 3, 6, and 12 months after radioactive I-125 seed-based stent implantation, the two groups of cholangiocarcinoma patients were reviewed, with the detection indicators including total bilirubin, tumor markers, P53, and biliary patency time measured through the CT or MRI. A total of 3 mL of venous blood was collected from the patients to measure the levels of the tumor markers CA19-9, CA242, and CEA. Additionally, the cholangiocarcinoma patients received percutaneous liver puncture for biliary tissue samples. Then, the tissue samples were subjected to Western blotting, during which they were incubated with the goat anti-human P53 monoclonal antibody at 37°C for 1–2 h, and washed using PBST for 3 times (5 min/time) and the expression level of P53 was measured finally.

### 1.5 Statistical methods

SPSS 22.0 software was used for statistical analysis. Enumeration data were expressed as mean ± standard deviation, and *t* test was performed for the bilirubin, tumor size, biliary patency time, levels of tumor markers and P53. The survival curve was plotted using GraphPad Prism 5, and *p*< 0.05 denoted that the differences were statistically significant.

## 2. Results

In this study, patients with cholangiocarcinoma selected for treatment in our hospital were treated with i125 seed stent implantation combined with TACE treatment. Observe the patient’s bilirubin level, tumor size change, biliary patency time, tumor marker level, correlation between tumor marker level and cholangiocarcinoma staging, P53 level, and patient prognosis before and after treatment. Furthermore, the effects of i125 seed stent implantation combined with TACE on tumor markers, P53 expression, and prognosis of patients with cholangiocarcinoma were analyzed.

### 2.1 Comparison of bilirubin level between before and after treatment

Before treatment, there was no difference in the bilirubin level between the two groups of patients. At 3 months after treatment, Group A had a higher level of bilirubin than group B (*p*< 0.05), and at 6 and 12 months after treatment, the level of bilirubin in Group A was slightly higher than that in Group B, with no statistically significant difference (*p*> 0.05). Moreover, the level of bilirubin was lowered in the two groups of patients after treatment (*p*< 0.05) (Table 2).

**Table 2.** Bilirubin changes before and after treatment

bilirubin	Group A	Group B	*t*	*p*
Before treatment	236.16 Voxel changes compared to	237.19 Voxel changes compared to	1.013	0.314
3 months after treatment	50.13 The vegan variation profile ^a^	31.04 The vegan variation profile ^a^	0.724	0.003
6 months after treatment	29.15 The vegan variation profile ^ab^	28.49 The vegan variation profile ^ab^	0.296	0.801
12 months after treatment	30.99 The vegan variation profile ^ab^	28.81 The vegan variation profile ^ab^	0.314	0.720

### 2.2 Comparison of tumor size between the two groups of patients before and after treatment

Before treatment, the tumor size was not statistically significantly different between the two groups of patients (*p*> 0.05). At 3 months after treatment, group A had slightly more lesions than Group B, without statistically significant difference (*p*> 0.05), while Group B exhibited substantially fewer lesions at 6 and 12 months after treatment (*p*< 0.05) (Table 3).

**Table 3.** Changes in tumor size (mm2) between the two groups

Tumor size	Group A mm^2^	Group B mm^2^	*t*	*P*
Before treatment	1567 Tumor size	1563 Tumor size	0.017	0.979
3 months after treatment	1293 ± 293	11,629 Tumor size	0.848	0.397
6 months after treatment	14,677 Tumor size ^a^	13,567 Tumor size	2.677	0.008
12 months after treatment	15,828 Tumor size ^ab^	14,358 Tumor size	3.503	<0.001

Tumor size: longest diameter × widest diameter/ mm^2^. a means pP<0.05 compared with before treatment, and b means pP<0.05 compared with 3 months after treatment.

### 2.3 Biliary patency time after treatment in the two groups of patients

**Figure 1.**
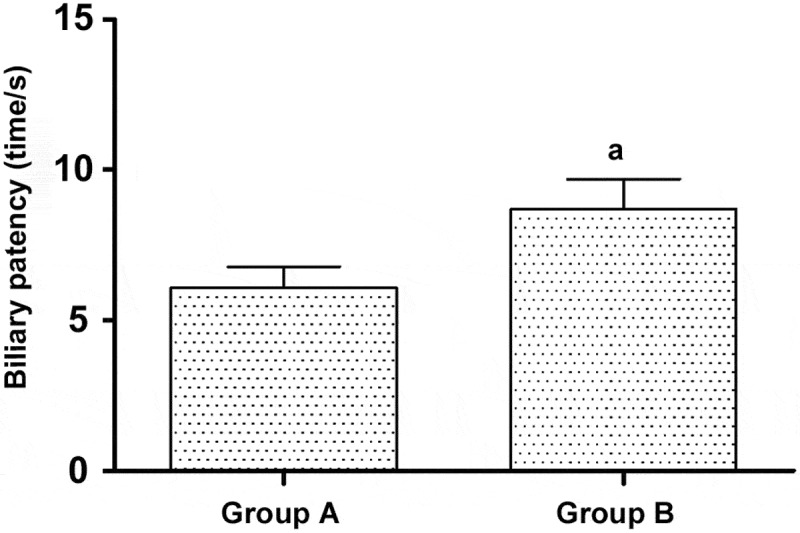
Comparison of biliary patency time after treatment between the two groups of patients.

After treatment, the biliary patency time in Group B was notably longer than that in Group A [(6.09 ± 1.21) s vs. (8.71 ± 1.17) s] (*p*< 0.05) (Figure 1).

a: p < 0.05 vs. Group A.

### 2.4 Correlations of tumor marker levels before and after treatment with cholangiocarcinoma stage

Before surgical treatment, the differences in the tumor markers were analyzed among 78 cholangiocarcinoma patients based on the pathological stage, and it was found that the patients at stage IIIhad substantially higher levels of CA19-9, CA242, and Cathan those at stages I–II (*p*< 0.05), suggesting that the cholangiocarcinoma stage is positively proportional to the levels of tumor markers (Table 4).

**Table 4.** The expression of tumor markers in stage I-stage patients before treatment

Cholangiocarcinoma staging	CA19-9(U/mL)	CA242(U/mL)	CEA(U/mL)
stageI	462.439 In direct proportion,	58.9739 In direct proportion,	42.1439 In direct proportion.15
stageII	594.729 In direct proportion, ^a^	65.1329.1^a^	57.0329 In direct proportion.15^a^
stageIII	779.399 In direct proportion, ^ab^	77.1199.15^ab^	68.9999 In direct proportion.15^ab^

CA19-9: Carbohydrate Antigen 19–9, Carbohydrate Antigen 19–9; CA242: Carbohydrate Antigen242; CEA: Carcinoembryonic antigen. A represents P < 0.05 compared to pretreatment and B represents P < 0.05 compared to 3 months after treatment.

### 2.5 Comparisons of tumor marker levels between the two groups of patients after treatment

Before treatment, there were no obvious differences in the levels of tumor markers between the two groups of patients (*p*> 0.05). After treatment, the levels of tumor markers substantially declined (*p*< 0.05), and Group B had lower expression levels of CA19-9, CA242, and CEA than Group A, with statistically significant differences (*p*< 0.05) (Table 5).

**Table 5.** Comparison of tumor marker expression after treatment between the two groups

Tumor markers	Group A	Group B	*t*	*P*
Before treatment				
CA19-9(U/mL)	608.27 Tumor markers	614.47 Post tumor markers	0.029	0.864
CA242(U/mL)	65.887 Tumor markers	67.127 Post tumor markers	0.035	0.862
CEA(U/mL	56.037 Tumor markers	58.127 Post tumor markers	0.042	0.713
3 months after treatment				
CA19-9(U/mL)	390.24 Tumor markers ^a^	293.42 Tumor markers ^a^	1.412	0.001
CA242(U/mL)	42.1442 Tumor markers ^a^	36.9642 Tumor markers ^a^	0.915	0.049
CEA(U/mL	40.222Tumor markers ^a^	37.022 Tumor markers^a^	1.015	0.036
6 months after treatment				
CA19-9(U/mL)	486.15 Tumor markers ^ab^	328.11 Tumor markers ^ab^	0.921	0.041
CA242(U/mL)	53.6911 Tumor markers ^ab^	45.7511 Tumor markers ^ab^	0.885	0.062
CEA(U/mL	49.2311Tumor markers ^ab^	41.1911 Tumor markers^ab^	0.912	0.049
12 months after treatment				
CA19-9(U/mL)	584.46Tumor markers ^bc^	512.43 Tumor markers ^abc^	1.031	0.031
CA242(U/mL)	67.2443Tumor markers ^bc^	54.1743 Tumor markers ^abc^	0.972	0.211
CEA(U/mL	55.3243Tumor markers ^bc^	49.7243 Tumor markers ^abc^	0.865	0.082

### 2.6 Expression level of P53 after treatment

Before treatment, there was no statistically significant difference in the expression level of P53 between the two groups of patients (*p*> 0.05), and the expression level was notably lowered at 3 months after treatment compared with that before treatment (*p*< 0.05) and markedly lower in Group B than that in Group A at 3, 6 and 12 months after treatment (*p*< 0.05) (Table 6).

**Table 6.** Comparison of p53 expression after treatment between the two groups

P53	Group A	Group B	*t*	*P*
Before treatment	1.75 Posttreatment, pretreatment	1.78 Posttreatment, pretreatment	0.638	0.913
3 months after treatment	1.233 Posttreatment, pretreatment ^a^	0.813 Posttreatment, pretreatment ^a^	7.152	0.026
6 months after treatment	1.676 Posttreatment, pretreatment ^b^	1.216 Posttreatment, pretreatment ^ab^	8.511	0.018
12 months after treatment	1.798 Posttreatment, pretreatment ^b^	1.358 Posttreatment, pretreatment ^ab^	6.152	0.046

### 2.7 Comparison of prognosis between the two groups of cholangiocarcinoma patients

**Figure 2.**
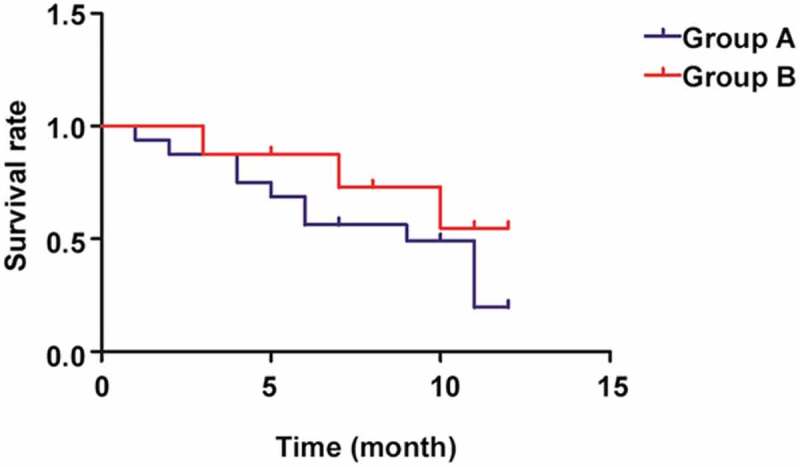
Patient survival in the two groups.

According to the comparison of survival rate between the two groups of patients (Figure 2), the 1-year survival rate in Group B was notably higher than that in Group A, showing a statistically significant difference (*p*< 0.05).

## 3. Discussion

Cholangiocarcinoma has no obvious symptoms at the early stage, and the majority of cases have been in the advanced stage when detected with a complex treatment process and a postoperative survival rate of only 10% [7]. As the understanding of this disease has been improved, and the medical imaging diagnosis techniques and excision surgery have been progressing in recent years, great improvements have been made in the clinical treatment of cholangiocarcinoma for which the only efficacious solution is surgical resection [8,9]. However, 80% of the patients have missed the best time for surgery, since there are no specific manifestations prior to operation. As a result, less than 50% of the patients undergo radical resection. Despite a certain advance in this cancer, the postoperative treatment outcomes are far from satisfactory, with a stubbornly low survival rate [10,11], so that surgical resection adds to the burden on the patients. Recent clinical treatments of cholangiocarcinoma have showed a certain efficacy, in which it is mainly the radioactive I-125 seed-based stent and TACE that play a protective role. Therefore, finding a novel safe and effective treatment for cholangiocarcinoma becomes the focus of research scholars at home and abroad.

The I125 radio particle stent belongs to the category of brachytherapy and has many advantages, such as low energy, long half-life, easy protection, no obvious radiation injury to healthcare workers and patients’ families, and is widely used in the clinic. Bonbon Chen et al 11 found that in patients with advanced gallbladder cancer complicated by obstructive jaundice who were inoperable as well as unwilling to receive radiotherapy and chemotherapy, the I125 radio particle stent had better efficacy and could obviously alleviate the clinical manifestations and improve the quality of patient life. TACE is one of the common treatment modalities for HCC patients who cannot be treated by surgical resection, which can effectively prolong the life cycle of patients and improve the prognosis of patients. Scoggins C R et al [12] study found that TEC had a role in gallbladder cancer treatment. The I125 radio particle scaffold and TACE play a protective role in the treatment of cholangiocarcinoma, and achieve a certain effect in the treatment of cholangiocarcinoma in the clinic. However, the effect of TECE combined with i125 radioactive particle stent on tumor markers, P53 levels and prognosis of patients with cholangiocarcinoma is still unclear.

According to the results of the present study, the bilirubin level was not different between the two groups of patients before treatment, and it was higher in Group A than that in Group B at 3 months after treatment (*p*< 0.05) but showed no statistically significant difference between the two groups of patients at 6 and 12 months after treatment (*p*> 0.05). Moreover, the level of bilirubin was lowered in the two groups of patients after treatment (*p*< 0.05). The tumor size did not differ notably between the two groups of patients before treatment and at 3 months after treatment (*p*> 0.05), and Group B had notably fewer lesions than Group A at 6 and 12 months after treatment (*p*< 0.05). Additionally, the mean patency time in Group B was remarkably longer than that in Group A (*p*< 0.05). Therefore, I-125 seed-based stent implantation combined with TACE is significantly efficacious for treatment of cholangiocarcinoma, which effectively lessens tumor lesions and reduces bilirubin. The study of Finkelmeier et al. approved that the role of I-125 seed-based stent implantation combined with TACE has been affirmed in treating malignant solid tumors, especially liver cancer [13]. Several studies have proposed that I-125 seed-based stent implantation combined with TACE makes notable contributions to the treatment of cholangiocarcinoma, and it extends the survival and life expectancy of the patients. Additionally, biliary stent implantation combined with TACE is efficacious to a certain degree for primary intrahepatic cancer accompanied by high-grade biliary obstruction [14]. This study further analyzed the effects of i125 seed stent implantation combined with TACE on tumor markers, P53 levels, and prognosis in patients with cholangiocarcinoma. CA19-9, CA242, CEA, and other tumor markers and P53 levels can be used as important indicators to evaluate the patient’s condition and prognosis, and provide an important theoretical basis for clinical evaluation and treatment of patients’ prognosis.

In the present study, the differences in the tumor markers were evaluated among 78 cholangiocarcinoma patients based on the pathological stage before operation, and it was found that the patients at stage III had substantially higher levels of CA19-9, CA242, and CEA than those at stages I–II (*p*< 0.05). Before treatment, the two groups of patients were not obviously different in the expression levels of tumor markers and P53 in biliary tissues (*p*> 0.05). The levels of tumor markers were dramatically lowered after treatment (*p*< 0.05), and Group B had lower expression levels of CA19-9, CA242, and CEA than Group A at 3, 6 and 12 months after treatment, with statistically significant differences (*p*< 0.05). Moreover, at 3 months after treatment, the expression level of P53 in the biliary tissues of patients was notably lower than that before treatment (*p*< 0.05), and it was remarkably lower in Group B than that in Group A at 3, 6 and 12 months after treatment (*p*< 0.05). A study demonstrated that the levels of serum tumor markers are highly valuable for the clinical evaluation of efficacy and prognosis in patients undergoing cancer surgery [15]. Besides, the expression level of P53 determines the clinical evaluation of the severity of cholangiocarcinoma in patients [16]. P53 can promote the apoptosis of tumor cells, thus alleviating diseases [17]. It was reported that I-125 seed-based stent implantation combined with TACE can lower the levels of tumor markers in the body of patients, which has an important implication for treatment of tumors [18]. In this study, it was found through the comparison of survival rate between the two groups of patients that the 1-year survival rate in Group B was notably higher than that in Group A, with a statistically significant difference (*p*< 0.05). Some studies have confirmed that I-125 seeds can be present for many purposes, in numerous forms and by diverse methods, and their combination with various treatment means can comprehensively treat hepatic, biliary, and pancreatic malignancies, and prolong the survival of patients [19,20], which is consistent with the results of this study.

## 4. Conclusion

I-125 seed-based stent implantation combined with TACE can effectively lower the levels of tumor markers and P53, shrink lesions and raise the survival rate in the patients, so it is of great significance for treatment of tumor.

## Data Availability

The datasets used and/or analyzed during the current study are available from the corresponding author on reasonable request.
